# Relationship of YKL-40 and adiponectin and subclinical atherosclerosis in asymptomatic patients with type 1 diabetes mellitus from a European Mediterranean population

**DOI:** 10.1186/s12933-015-0287-z

**Published:** 2015-09-18

**Authors:** Eva Aguilera, Enric Serra-Planas, M. Luisa Granada, Silvia Pellitero, Jordi L. Reverter, Núria Alonso, Berta Soldevila, Dídac Mauricio, Manel Puig-Domingo

**Affiliations:** Endocrinology and Nutrition Unit, Department of Medicine, Institute Research and Hospital Germans Trias i Pujol, Universitat Autònoma de Barcelona, Crta. Canyet s/n, 08916 Badalona, Barcelona Spain; Centro de Investigación Biomédica en Red de Diabetes y Enfermedades Metabolicas Asociadas (CIBERDEM), Instituto de Salud Carlos III (ISCIII), Madrid, Spain; Biochemistry Unit, Hospital Germans Trias i Pujol, Badalona, Barcelona Spain

**Keywords:** YKL-40, Adiponectin, Subclinical atherosclerosis, Type 1 diabetes

## Abstract

**Background:**

The glycoprotein YKL-40 is a new marker of early inflammation and endothelial dysfunction. Adiponectin is a collagen-like protein with anti-atherogenic and anti-inflammatory effects. Increased concentrations of both markers have been reported in patients with type 1 diabetes (T1D).

**Aim:**

To assess the possible role of YKL-40 and adiponectin as a marker of subclinical cardiovascular disease in asymptomatic patients with type 1 diabetes with no history of ischemic or macrovascular heart disease and its relationship with other classic inflammatory biomarkers.

**Methods:**

Concentrations of YKL-40, adiponectin, IL-6, IL-1β, TNF- α, hsCRP and homocysteine were determined in 150 T1D patients (58 % men, age: 38.6 ± 8.1 years, 20.4 ± 8.1 years of evolution, BMI: 25.1 ± 3.6 kg/m^2^; HbA_1c_ 8.1 ± 2.3 %, 48 % smokers; 26 % retinopathy, microalbuminuria 9 %) and 50 controls age, sex and smoke condition matched. Subclinical atherosclerosis was assessed by a carotid ultrasonography and a computed tomography for evaluation of calcium artery calcification score (CACS).

**Results:**

82 % of T1D patients and 92 % of controls had a calcium score of 0. T1D patients showed a significantly higher mean common carotid artery intima media thickness (CIMT) compared to controls (0.55 ± 0.14 vs 0.48 ± 0.14 mm, p = 0.01). Concentrations of YKL-40 and adiponectin were significantly higher in T1D [42.6 (10.4–195.0) vs ±28.7 (11.0–51.2) ng/ml, p = 0.001 and 15.8 ± 9.1 vs. 12.4 ± 5.3 mg/ml, p = 0.008], with no differences when compared to other inflammatory parameters. In T1D patients no association was found between YKL-40 and adiponectin and screening test for subclinical arterial disease (neither CACS nor CIMT). A positive correlation was found between levels of YKL-40 and age and duration of disease (r = 0.28, p = 0.003; r = 0.35, p = 0.001). There were no differences in the YKL-40 in relation to the presence or absence of retinopathy or nephropathy. Levels of adiponectin were higher in patients with nephropathy (21.84 ± 8.15 vs. 14.88 ± 8.27 mg/ml, p = 0.008).

**Conclusions:**

Type 1 diabetes patients from a Mediterranean area with a longer disease evolution, although a lower degree of subclinical disease, showed significatively higher concentrations of YKL-40 and adiponectin compared with the controls. Therefore, we conclude that YKL-40 and adiponectin are early inflammatory markers in diabetic subjects even in the presence of a low atherosclerotic background.

## Background

Type 1 diabetes mellitus (T1D) is associated with an increase in cardiovascular disease and higher mortality compared to the non-diabetic population [1, 2]. Different studies have demonstrated that functional and structural vascular changes as altered endothelial function occur at a very early stage of the course of T1D [3–5].

In a similar way that for type 2 diabetes (T2D), some authors have proposed that silent subacute inflammation is also operative in T1D as a driver of cardiovascular disease development. Thereby, it has been reported that low adiponectin levels predict cardiovascular events and coronary artery calcification (CAC), as well as levels of soluble interleukin 2 receptor correlate also with CAC progression [6–8].

Recently, the possible role in atherosclerosis and diabetes of new inflammatory markers as YKL-40 has been investigated. YKL-40 is a 40 kDa heparin- and chitin-binding glycoprotein, which is expressed and secreted by different cell types of the immune system, differentiated vascular endothelial and smooth muscle cells. This inflammatory glycoprotein seems to play an important role in endothelial dysfunction and during the early stages of atherosclerosis [9]. Several studies have reported elevated YKL-40 levels in different cardiovascular conditions and it has been described a positive association between this marker and mortality [10–17]. It has been reported an increased YKL-40 expression in patients with carotid atherosclerosis [18], as well as a positive correlation between common carotid artery intima media thickness (CIMT) and YKL-40 levels in patients with obstructive sleep apnea syndrome [16] and between arterial stiffness and YKL-40 levels in essential hypertension subjects [15]. Increased YKL-40 levels have also been found both in patients with T1D and T2D [19–22], and particularly in T1D and T2D patients with microalbuminuria, suggesting that this glycoprotein might be used as an early marker of vascular disease [18, 21]. A recent study has reported higher levels of YKL-40 in asymptomatic T2D patients with suspected coronary artery disease (CAD) suggesting a possible association between this marker and CAD [23].

Adiponectin is a collagen-like protein secreted from the adipose tissue and it has anti-atherogenic and anti-inflammatory effects. Hypoadiponectinemia have been reported associated with obesity, metabolic syndrome, atherosclerosis, CAD and atherogenic dyslipidemia with low-density lipoprotein (LDL) subfraction phenotype, as well as in patients with T2D [24, 25]. Nevertheless, higher adiponectin levels have been described in T1D patients and have been associated with impairment of renal function and longer evolution disease. Despite these higher levels and the anti-atherogenic effects of adiponectin, there is an increase in the cardiovascular mortality in T1D patients [6, 26–29].

There is scarce information concerning the possible role of adiponectin as an indicator of subclinical atherosclerosis. A recent study has shown no correlation between adiponectin and CIMT in children with T1D [30].

There are no studies evaluating the association between YKL-40 with subclinical CAD in T1D patients. Therefore, the aim of this study was to assess the possible role of YKL-40 and adiponectin as a marker of subclinical cardiovascular disease in asymptomatic patients with T1D from a Mediterranean area and its potential relationship with other classic inflammatory biomarkers.

## Methods

An initial cohort of 150 asymptomatic T1D consecutively followed at our outpatient clinic and a control group of 50 subjects matched by age, sex and smoke condition were recruited between 2010 and 2012 in Badalona, Barcelona. In this cohort we evaluated the presence of subclinical atherosclerosis as previously reported [31]. Inclusion criteria were an age between 20 and 50 years and an evolution disease of more than 10 years. The exclusion criteria were a previous history of clinical macrovascular or cardiac heart disease (CHD). Current smoking and previously smoking condition for less than 5 years were included in the same category. A group of non-diabetic subjects matched for age, sex, body mass index (BMI) and smoking condition recruited from the relatives and staff of our hospital was also included as control group. All patients were under intensive insulin treatment and 15 % of them using pump devices.

The local ethics committee, in accordance with the Declaration of Helsinki, approved the study; all participants gave their written informed consent prior to inclusion.

Demographic and clinical data including age, sex, history of clinical macrovascular disease and microvascular diabetic complications, family history of early CHD in first degree relatives (defined as CHD occurring before age 55 years in men and before age 65 years in women) and medical treatment (antihypertensive agents, statins and acetylsalicylic acid) were recorded for all patients. BMI was calculated as weight in kilograms divided by height per square meter.

Diabetic nephropathy was evaluated according to urinary albumin excretion. Thus, normal urinary albumin excretion was considered below 30 mg/24 h, microalbuminuria from 30 to 300 mg/24 h and proteinuria above 300 mg/24 h. These results were confirmed on at least two out of three consecutive determinations. Diabetic retinopathy was defined by fundus oculi performed by a specialized ophthalmologist.

Trained personnel collected clinical parameters (age, sex, height, weight, BMI, blood pressure and smoking habit and family history of early CHD).

### Biochemical measurements

Blood samples were drawn by venipuncture at between 8.00 and 08.30 h after an overnight fast. Plasma glucose, total cholesterol, high density (HDL) and low density (LDL) lipoprotein cholesterol, triglycerides, calcium (Ca) and phosphate (Ph) were measured by routine clinical chemistry immediately after extraction. HbA1c was measured in blood samples with ethylenediaminetetraacetic acid (EDTA) by high-performance liquid chromatographic (HPLC) using a fully-automated Adams Menarini HI-AUTO A1c 8160 analyzer manufactured by Arkray (Kyoto, Japan) with an inter-assay coefficient of variation of 1.8 and 1.5 % at HbA1c levels of 4.8 and 9.0 % respectively (reference range 4–5.8 %). This method is a cation exchange HPLC method certified by the NGSP (National Glycohemoglobin Standardization Program) of traceability to the Diabetes Control and Complications Trial Reference (DCCT) Method. Mean HbA_1c_ was calculated as an average of three determinations in the previous year before the inclusion in the study.

Serum YKL-40 was analyzed using a commercial ELISA assay (Quidel Speciality products, San Diego, CA USA), Sensitivity of the assay was 20 μg/ml. The average within-run and total CVs were 3.6 and 5.4 %, respectively. The reference interval (central 90 % interval) for chondrex in healthy adults was 25–95 μg/l.

Serum adiponectin concentrations were measured by a commercial radioimmunoassay (Linco Research, Inc., St Louis. MO, USA), using ^125^I-labeled technique. The intra- and inter-assay coefficients of variation (CVs) at mean adiponectin levels of 3, 6 and 15 μg/ml were 3.6, 6.2 and 1.8 % intra-assay, and 9.2, 6.9 and 9.2 % inter-assay, respectively. Assay sensitivity was 1 μg/ml. All plasma samples were diluted 1:250 yielding an effective range of 0.2–50 μg/ml.

Plasma homocysteine concentrations were measured using an automated enzyme chemiluminescence immunoassay (Immulite 2000^®^ Siemens Healthcare Diagnostics, Llanberis, UK). Assay sensitivity was 1.2 μmol/l. The intra- and inter-assay coefficients of variation (CVs) at mean homocysteine concentrations of 11 and 24.5 μmol/l were 8.1 and 5.6 % intra-assay, and 8.5 and 8.1 % inter-assay, respectively (reference range 5.0–12 μmol/l).

Serum TNF- α, IL-6 and IL-1β concentrations were measured using an enzyme chemiluminescence immunometric assay (Immulite^®^, Siemens Healthcare Diagnostics, Llanberis, UK). For TNF-α, analytical sensitivity was 1.7 pg/mL and interassay CV <6.8 %. For IL-6 analytical sensitivity was 2 pg/ml and interassay CV <7.3 %. For IL-1β analytical sensitivity was 1.5 pg/ml and interassay CV <9.1 %.

Serum high sensitivity C-reactive protein (hsCRP) were measured using an ultrasensitive CRP test (N High Sensitivity CRP) on a BN-ProSpec nephelometer (Dade Behring, GMBH, Marburg, Germany) with an inter-assay variation coefficient of 3.7 and 3.5 % for CRP concentrations of 2.38 and 52.2 mg/l, respectively. Assay was 0.175 mg/l, performed using a sample dilution of 1:20.

### Evaluation of subclinical atherosclerosis

A computed tomography (CT) to quantify coronary artery calcification score (CACS) was performed using a 16-slice high resolution CT ECG-gated, with retrospective reconstruction and with special attention to the coronary arteries (SOMATOM Sensation 16 and Syngo Calcium Scoring software for analysis and calcium calcification). CACS was identified as a dense area in the coronary artery exceeding the threshold of 130 Hounsfield units. A total Agatston score was determined for each patient. The results were expressed according to the classification previously described by Shaw et al. [32] and results were transformed as a categorical variable considering positive those greater than zero and negative those with negative results equal to zero.

A carotid ultrasound (CU) to measure the CIMT was performed in all participants. Ultrasonographic images were acquired using high resolution B-mode ultrasound (Siemens Acuson Sequoia 512) with an electric linear array 13-5 MHz transducer. The CIMT was the result of the median of the tunica intima and tunica media thickness in per protocol defined carotid areas (left internal carotid, right internal carotid, common carotid and bifurcation). Plaque number and characteristics were recorded.

A single trained radiologist performed evaluation and acquisition of CT images and CU.

### Statistical methods

Continuous variables were expressed as mean standard deviations (SD) or median (percentile 2.5 and 97.5) and categorical variables as frequency and/or percentage. The Student’s t-test or the non-parametric Mann–Whitney U test, as appropriated, tested differences between groups. A p value less than 0.05 was considered statistically significant. Categorical variables were compared with a χ^2^ test. Correlation analyses between continuous variables were performed by using univariant Spearman correlation analysis. All statistical analyses were performed using the Statistical Package for Social Science (SPSS, Chicago, IL, USA) for personal computers, version 12.0 (SPSS).

## Results

### Subclinical atherosclerosis evaluation

#### Carotid ultrasonography

Type 1 diabetes patients showed a significantly higher common carotid artery intima-media thickness (CIMT) compared to control group (0.55 ± 0.14 vs 0.48 ± 0.14 mm, p < 0.01). There were no differences in the number of plaques between patients and controls. A low proportion of subjects in both groups presented atheroma plaques (16 patients and 4 control subjects, 11 vs 8 %), in all cases conditioning stenosis of less than 50 %.

#### Carotid calcification

A high proportion of subjects in both groups, patients and controls, displayed a CACS of 0 (92 vs 82 %), and only 24 T1D patients and four controls presented a calcium score greater than 0. The differences between both groups were not significant (16.6 vs 8 % in controls, p = 0.236).

### YKL-40 and inflammatory markers

**Fig. 1:**
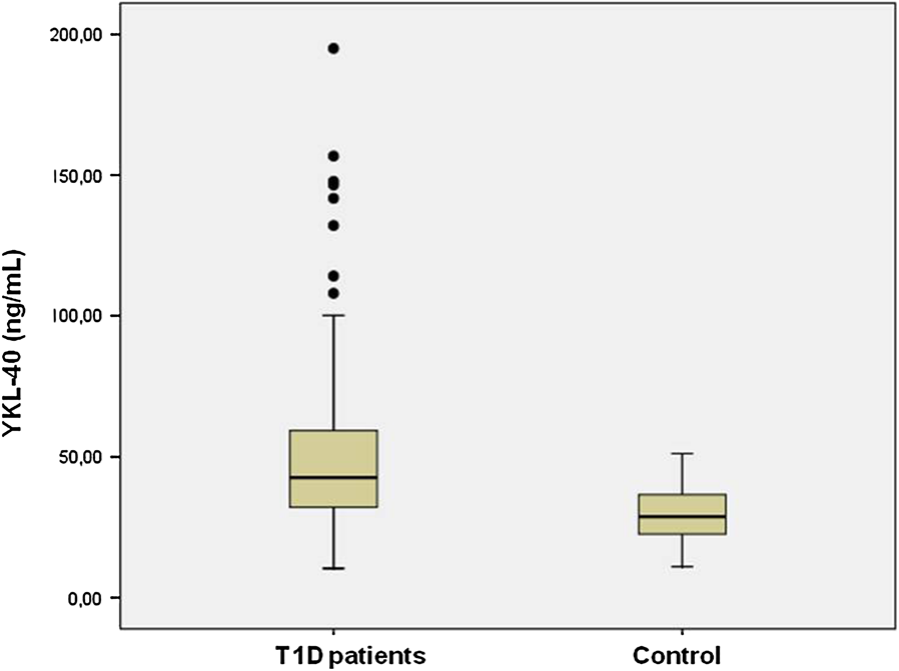
Comparison of serum YKL-40 levels between patients with type 1 diabetes patients and control group. Data are median levels (range)

The baseline characteristics of the whole sample and the biomarkers levels are shown in Table 1. Levels of YKL-40 were significantly higher in T1D than in controls [42.6 (10.4–195.0) vs ±28.7 (11.0–51.2) ng/ml p = 0.001] (Fig. 1) and levels of adiponectin were also significantly higher in T1D patients (15.8 ± 9.1 vs 12.4 ± 5.3 mg/ml, p = 0.008), with no differences between patients and controls in the other inflammatory parameters.

**Table 1:** Basal characteristics and biomarkers levels of patients with type 1 diabetes and control group

	Type 1 diabetes	Control	p
N	150	50	
Age (years)	38.6 ± 8.1	38.1 ± 7.2	NS
Sex (male/female %)	58/42	56/44	NS
Evolution type 1 diabetes (years)	20.4 ± 8.1	–	–
Family history CHD (%)	20	22	NS
Body mass index (kg/m^2^)	25.1 ± 3.6	25.3 ± 4.3	NS
Systolic blood pressure (mmHg)	117.3 ± 13.5	–	–
Diastolic blood pressure (mmHg)	70.9 ± 7.7	–	–
Retinopathy (%)	26	–	–
Nephropathy (%)	9	–	–
HbA1c (%, mmol/mol)	8.1 ± 2.3 (64.9 ± 11.4)	–	–
Total cholesterol (mg/dl)	182.7 ± 25.1	191.1 ± 34.1	NS
HDL (mg/dl)	60.3 ± 15.1	61.8 ± 16.6	NS
LDL (mg/dl)	105.3 ± 21.9	111.3 ± 33.5	NS
Smoke (% yes/no)	48/52	42/58	NS
Statins (%)	21	–	–
Antihypertensive (%)	15	–	–
Acetylsalicylic acid (%)	16	–	–
YKL-40 (ng/ml)	42.6 (10.4–195.0)	28.7 (11.0–51.2)	0.001
Adiponectin (mg/ml)	15.4 ± 8.4	11.9 ± 5.1	0.007
TNF-α (pg/ml)	7.5 ± 2.4	7.8 ± 1.8	NS
IL-1β (pg/ml)	1.0 ± 1.1	1.1 ± 0.8	NS
IL-6 (pg/ml)	2.0 ± 9.5	3.0 ± 4.0	NS
Homocysteine (mmol/l)	9.74 ± 5.1	10.9 ± 3.4	NS
hsCRP (mg/l)	2.21 ± 3,9	2.5 ± 3.9	NS

#### YKL-40, adiponectin and subclinical atherosclerosis imaging

When univariate correlation analyses were performed, we did not find association between YKL-40 and adiponectin levels and any cardiovascular surrogates, neither CACS nor CIMT, either when these variables were evaluated as continuous or categorical variables (presence of carotid plaques and CACS 0 or ≥1). There were no differences between the mean YKL-40 levels of subjects with a CACS of 0 and those with a CACS greater than 0. When T1D were categorized according to the presence of plaques there were no differences.

There were no positive correlations of the other inflammatory markers evaluated (TNF-α, IL-6, IL-1β, homocysteine, hsCRP) in relation to the imaging markers of subclinical atherosclerosis.

#### YKL-40, adiponectin, age and evolution of T1D

A positive correlation was found between levels of YKL-40 and age and duration of disease (r = 0.28, p = 0.003; r = 0.35, p = 0.001). Levels of adiponectin were also positively correlated with age (r = 0.25, p = 0.01). However, no correlations were found between YKL-40 and adiponectin.

#### YKL-40, cardiovascular risk factors and metabolic control

Patients under treatment with antihypertensive agents displayed significantly higher levels of YKL-40 [41.4 (10.41–195.1) vs 28.8 (11.0–51.2) ng/ml, p = 0.04] compared to those patients without treatment.

Levels of YKL-40 were not found different regarding gender, BMI, smoking status, treatment with statins or acetylsalicylic acid and HbA_1c_ values.

#### YKL-40, adiponectin and microvascular complications

There were no differences in YKL-40 concentrations in relation to the presence or absence of retinopathy or nephropathy. Levels of adiponectin were higher in patients with nephropathy (21.84 ± 8.15 vs 14.88 ± 8.27 mg/ml, p = 0.008), but not regarding the presence of retinopathy; neither TNF- α, nor IL-6 and IL-1β showed differences regarding the presence of microvascular disease.

A positive correlation was found between the concentrations of YKL-40 and TNF-α (r = 0.24, p = 0.02), but not with the other inflammatory markers evaluated. IL-1β and IL-6 and TNF-α correlated among them (r = 0.39, p = 0.001; r = 0.36, p = 0.001, respectively).

## Discussion

In this cohort of patients leaving in a Mediterranean area and with a relatively long disease evolution we did not find an association between YKL-40 and adiponectin levels and the presence of subclinical CAD evaluated by CACS and CIMT. However, our T1D patients displayed higher levels of YKL-40 than control subjects. These findings are in concordance with two previous studies performed in T1D [19, 20]. Rathcke et al. [20] reported elevated YKL-40 levels in a cohort of Danish, middle-aged (50 years old) T1D patients, with a long diabetes duration (30 years) and with a high prevalence of microvascular complications (more than 50 %). Sakamoto et al. [19] showed also elevated levels of YKL-40 in a cohort of Japanese T1D patients younger than the cohort of Danish patients (mean age of 25 years), with less mean duration of diabetes (13 years) and with a lower prevalence of microvascular complications (9 % of microalbuminuria, 33 % of retinopathy). Our cohort of T1D showed a mean age and duration of the disease intermediate between these two specific studies, but with a much lower prevalence of microangiopathy than the Danish cohort and similar to the Japanese patients. Despite that these two studies have described elevated concentrations of YKL-40 in T1D patients with microalbuminuria, it was not the case in our patients, indicating that YKL-40 is already increased even at a stage when subclinical micro and macro vascular disease is not detected with current clinical procedures or if it is modestly present. The low proportion of our T1D patients presenting nephropathy (9 %) but showing high YKL-40 compared to controls clearly indicates that at this stage of T1D evolution, inflammatory status is already activated. Moreover, we did not find higher levels of YKL-40 in nephropathy patients at the stage of positive microalbuminuria, and although we did not find differences according to the presence or absence of retinopathy, probably due to the low number of subjects included in our cohort, all the data taken together suggest that more prospective studies are probably needed in order to clarify if relevant changes in the magnitude of YKL concentration are detected over time and in relation to the appearance of microvascular disease.

Several clinical studies documented elevated YKL-40 levels in patients with cardiovascular disease, including CAD, peripheral artery disease and stroke, either with or the coexistence of diabetes. An association has also been described between higher YKL-40 levels and an increased mortality, especially in elderly persons with stable CAD [11–14]. Nevertheless, there are no studies yet aimed to determine the possible role of this glycoprotein in patients with T1D and cardiovascular disease. The prevalence of subclinical CAD was low in our cohort of asymptomatic T1D patients and we did not find association between YKL-40 levels and any cardiovascular surrogate, neither CACS nor CIMT, either when these variables were evaluated as continuous or categorical variables, thus suggesting that YKL-40 elevation is more a marker of increased inflammation in T1D patients, rather than one indicating subclinical disease. In our cohort, there was a minimal degree of overlap between control and patients, which seems to reflect that with a so low degree of subclinical disease in our diabetic cohort, inflammation is a state substantially linked to T1D before organ lesions are detectable, and which concentrations start to increase probably early after diagnosis. This finding is in accordance with the Japanese cohort, in which there was no significant correlation between YKL-40 levels and CIMT [19]. The only relationship we found was that in patients treated with antihypertensive drugs, YKL-40 was higher, although in those with established nephropathy there were no differences; we cannot at this point speculate whether these differences are due to the fact that those patients had a higher degree of vascular lesion or to the antihypertensive treatment itself. At the low level of subclinical CAD of our population when compared to the controls, there were no differences in the rest of circulating concentrations of classical inflammatory biomarkers studied between the two groups. Although we did not find correlation in this cross-sectional evaluation of YKL-40 with metabolic control—measured as A_1c_ levels—it will be interesting to study if YKL-40 concentrations are modified by intensification of metabolic parameters or in a broader range of A_1c_.

Our lack of correlation between adiponectin and CIMT is in concordance with a previous study conducted in children with T1D [30]. A positive correlation between higher concentrations of adiponectin and the presence of microalbuminuria was also found in this group of T1D patients. Also, this positive correlation has been reported by other authors [28, 29].

## Conclusions

In T1D patients from a Mediterranean area with a long disease evolution although a low degree of subclinical disease, we found significatively higher concentrations of YKL-40 and adiponectin compared with the controls. Therefore, we conclude that YKL-40 and adiponectin are early inflammatory markers in diabetic subjects with low atherosclerotic background. Further studies with a large number of patients will be necessary in order to evaluate the usefulness of changes in YKL-40 and adiponectin concentrations in progressive atherosclerotic diabetic disease, as well as its potential role as predictor biomarker of vascular disease in T1D.

## Abbreviations

T1D: type 1 diabetes mellitus
T2D: type 2 diabetes mellitus
CVD: cardiovascular disease
CIMT: common carotid artery intima media thickness
CAD: coronary artery disease
CHD: cardiac heart disease
CACS: coronary artery calcification
hsCRP: high sensitivity C-reactive protein
CT: computed tomography
BMI: body-mass
LDL: low-density lipoprotein
HDL: high-density lipoprotein
HbA_1c_: glycated hemoglobin
